# Hand Motion Analysis Illustrates Differences When Drilling Cadaveric and Printed Temporal Bone

**DOI:** 10.1177/00034894211059310

**Published:** 2021-12-07

**Authors:** Jordan B. Hochman, Justyn Pisa, Katrice Kazmerik, Bertram Unger

**Affiliations:** 1 Faculty of Health Sciences, University of Manitoba, Winnipeg, MB, Canada; 2 Department of Otolaryngology - Head and Neck Surgery, Faculty of Medicine, University of Manitoba, Winnipeg, MB, Canada; 3 Department of Otolaryngology - Head and Neck Surgery, Health Sciences Centre, Winnipeg, MB, Canada; 4 Department of Family Medicine, Pure Lifestyle, Winnipeg, MB, Canada; 5 Department of Medical Education, Faculty of Medicine, University of Manitoba, Winnipeg, MB, Canada

**Keywords:** temporal, bone, hand, motion, 3D, printed

## Abstract

**Objective:**

Temporal bone simulation is now commonly used to augment cadaveric education. Assessment of these tools is ongoing, with haptic modeling illustrating dissimilar motion patterns compared to cadaveric opportunities. This has the potential to result in maladaptive skill development. It is hypothesized that trainee drill motion patterns during printed model dissection may likewise demonstrate dissimilar hand motion patterns.

**Methods:**

Resident surgeons dissected 3D-printed temporal bones generated from microCT data and cadaveric simulations. A magnetic position tracking system (TrakSTAR Ascension, Yarraville, Australia) captured drill position and orientation. Skill assessment included cortical mastoidectomy, thinning procedures (sigmoid sinus, dural plate, posterior canal wall) and facial recess development. Dissection was performed by 8 trainees (n = 5 < PGY3 > n = 3) using k-cos metrics to analyze drill strokes within position recordings. K-cos metrics define strokes by change in direction, providing metrics for stroke duration, curvature, and length.

**Results:**

*T*-tests between models showed no significant difference in drill stroke frequency (cadaveric = 1.36/s, printed = 1.50/s, *P* < .40) but demonstrate significantly shorter duration (cadaveric = 0.37 s, printed = 0.16 s, *P* < .01) and a higher percentage of curved strokes (cadaveric = 31, printed = 67, *P* < .01) employed in printed bone dissection. Junior staff used a higher number of short strokes (junior = 0.54, senior = 0.38, *P* < .01) and higher percentage of curved strokes (junior = 35%, senior = 21%, *P* < .01).

**Conclusions:**

Significant differences in hand motions were present between simulations, however the significance is unclear. This may indicate that printed bone is not best positioned to be the principal training schema.

## Introduction

Mastery of temporal bone surgery is achieved through apprenticeship with adjunctive cadaveric experience. These opportunities permit skill acquisition and development of insight.^1^ Cadaveric temporal bone dissection has traditionally been the standard in surgical training.^1-3^ However, learning opportunities are progressively inaccessible, due to limited cadaveric availability as well as cost, social, and political considerations.^1-3^

The development of virtual technologies, haptics, and rapid prototype modeling may provide additional training opportunities.^4-9^ Simulations represent a real-world process that facilitates learning through immersion, reflection, feedback, and risk-free practice, while providing the capacity to shape graduated exposure to pathologic conditions.^8^ As competency-based medical education becomes increasingly prevalent, simulation provides a supplement to conventional operating room training. An opportunity that allows for the assessment of surgical skill^8-10^ and permits supplementary educational opportunities that do not risk patient safety.

Factors such as the fidelity of a temporal bone analog and the ability to recreate a realistic experience with these models is paramount.^1,11,12^ Both haptic and rapid prototype models have been shown to be capable of high levels of anatomical fidelity, however users of virtual haptic models often complain of unrealistic force feedback while drilling.^5,7,13,14^ A direct comparison between the 2 forms of simulation illustrated a strong end-user perception of better bone character and drill/bone interaction with the printed model.^15^

Significant differences in drill technique were found in a study by Ioannou et al^13^ assessing and contrasting virtual haptic and cadaveric temporal bone simulation. Drill strokes were significantly different across most stages of dissection and exhibited different patterns of dissection.^13^ There was a propensity toward straight strokes and fewer rounded strokes with virtual haptic simulations compared to cadaveric drilling. The study concluded that the user employed disparate drill technique between the 2 environments. The concern this raises is that while the anatomical fidelity is strong, there is the potential for the development of non-productive and unsafe surgical technique. This concern may extend to printed simulations as well, necessitating study of the motions and procedures used by those training with printed bone models.

A mechanism to undertake such an assessment can involves Hand Motion Analysis (HMA), employing an electromagnetic field to track sensor data and capture motions.^13,16^ Based on the data collected, various metrics such as time taken to complete a procedure, number of strokes, path of the dominant hand, acceleration, and velocity have all been used to quantify drill technique.^16-18^ Using HMA allows skill to be measured quantitatively by relating it to the subject’s dexterity and technique.^13,16^

Determining the similarities and differences between temporal bone surgical simulations will strongly influence the potential applications of these technologies. Models that teach accurate drilling technique alongside anatomical correctness and strong bone-like character can be used to supplement cadaveric opportunities. Sound educational theory precludes use of a tool that results in maladaptive skill development. The use of cadaveric specimens will remain essential to learning temporal bone anatomy and surgical technique, but the development of new modalities for surgical resident training permits metric assessments, standardization of the learning environment and deliberate practice.^1-3^

This study aims to compare drill technique via HMA during dissection of both cadaveric and printed bone models. The Laboratory for Surgical Modeling, Simulation, and Robotics has developed a printed model based on micro-computer tomography (CT) data and uses specific materials that mimic bone.^7,15^

## Methods

The study was comprised of eight (8) Otolaryngology—Head and Neck Surgery residents; 5 junior residents (PGY 1-3) and 3 senior residents (PGY 4, 5). Approval was granted by the University of Manitoba’s Office of Research Ethics & Compliance (H2019:103-HS22675).

Participants dissected both a cadaveric and a printed bone model with assignment to the initial condition, using computer generated randomization. The specific task was to complete a cortical mastoidectomy with posterior tympanotomy. Both the cadaveric and printed models were mounted in a cadaveric temporal bone bowl holder to mimic the surgical approach to a live patient, and also maintain a level of consistency. Each participant was asked to perform 3 different but well-defined stages while drilling: (1) cortical mastoidectomy, (2) thinning procedures including posterior canal wall thinning, drilling along the dural plate, and sigmoid sinus, and (3) drilling a facial recess.^13,19^ These divisions were created to separate the different techniques used during conventional dissection.^20,21^

### Development of the Printed Bone Model

**Figure 1.**
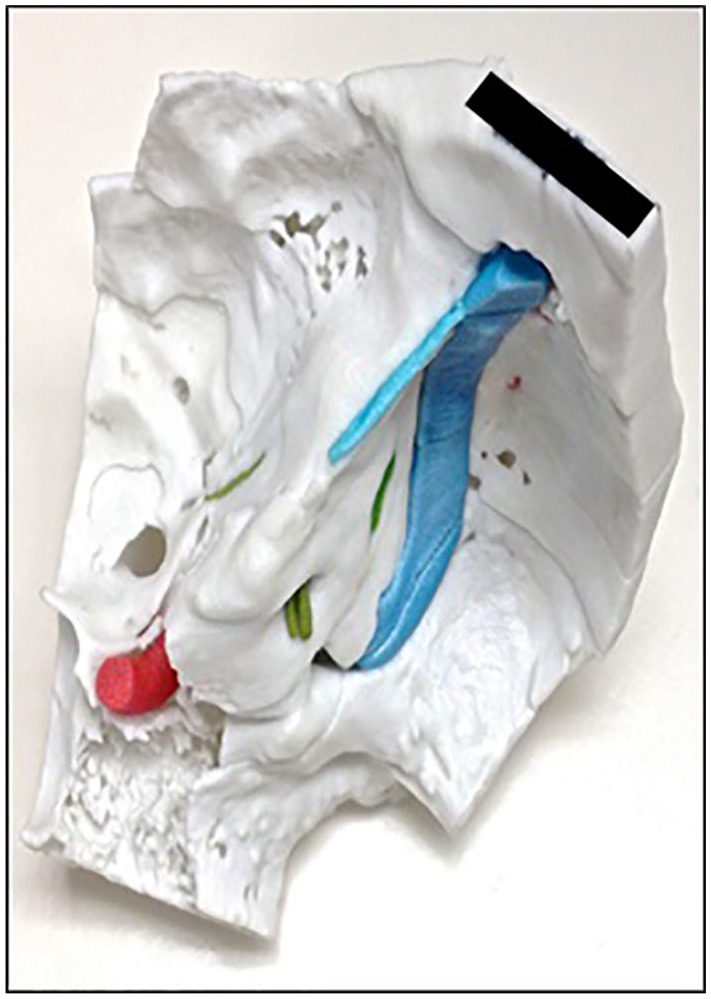
Completed 3D printed temporal bone model with internal anatomical structures. Identified are the carotid artery, sigmoid sinus, endolymphatic sac, superior petrosal sinus, greater superficial petrosal nerve, and IAC contents.

The process for generating the model has been previously published^7,15^ The prototype has internal fidelity with production requiring several steps. Volumetric CT images are segmented into anatomical regions of interest, each defined as distinct polygon meshes. These meshes are combined, voxellated, and sliced into sections for printing, after which alignment fiducials are added. Individual slices are then combined to produce a final physical model (Figure 1).

### Hand Motion Tracking and Analysis

**Figure 2.**
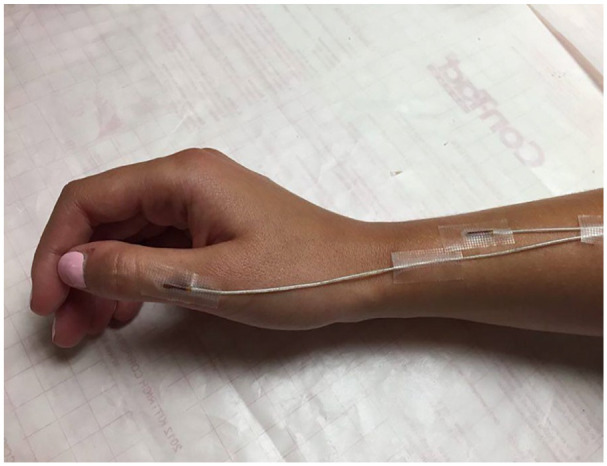
Hand sensor placement for dominant hand. Sensors are attached just below the radial styloid process, and above the first metacarpophalangeal joint or mid-thumb.

Electromagnetic motion tracking sensors (Ascension Trackstar, Yarraville, Australia) were used to capture the hand motions. Two larger (8 mm) sensors were placed on bowl-type holder and to the surgical drill to enable tracking the drill shaft relative to the model. Two smaller sensors (2 mm) were attached on the participants’ wrist, just below the radial styloid process, and a fourth was placed above the first metacarpophalangeal joint or mid-thumb (Figure 2). The small size of the sensors ensured that they did not hinder or alter trainees’ activities.

The motion tracking device recorded hand motion signals in terms of position measures on a Cartesian plane as well as orientation measures (azimuth, elevation, and roll) with the accuracy of 1.4 mm and 0.5° RMS for position and orientation measures, respectively. These data are displayed in real-time using Cube software. *T*-Test statistical analysis was used to determine statistically significant differences between drilling techniques employed on printed and cadaveric bone samples.

Motion recording may be influenced by electromagnetic interference from drill vibration. In order to avoid corrupted information, data were recorded in time segment of 5 minute or less. This minimized the recording of large repositioning movement as well as other movements unrelated to drilling technique.

### Software Calibration

Software was developed internally. Preliminary analysis was done using Motion Analysis and Recording Systems (MARS) software which converts *x, y*, and *z* position values into dynamic metrics such as velocity and acceleration. These metrics can then be used to define recorded motions as individual strokes based on filters and thresholds allowing for objective comparison between the cadaveric bone and printed models.

Thresholds were set to limit what is considered a meaningful movement made by the participant in an attempt to reduce hand tremor, drill vibrations, and experimental error. A high and low threshold for each metric was chosen based on previously validated values.^16,18^

When using velocity as a measure, both direction and speed are taken into account. Defining a stroke was determined using a change of velocity greater than 5 mm/s at the low threshold, while 15 mm/s was required for the higher threshold. When using acceleration to define a stroke, an acceleration of 2 mm/s^2^ was required at low threshold, while an acceleration of 5 mm/s^2^ was required for the higher threshold. Lower thresholds capture finer movements but allow more noise to pass. Higher thresholds decrease the amount of noise but lose finer motions. For this reason, both thresholds were taken into consideration during data analysis.

Further analysis was done using a second piece of internally developed software that uses a cosine (k-cos) function to detect changes in direction in order to identify strokes.^22^ This method of analysis parallels the analysis of Ioannou et al.^13^ Using this k-cos function, a stroke is determined based on directional change or the curvature within a frame of data points.^13^ A frame rate-independent low pass smoothing filter was used to eliminate vibrations from the drill, hand tremor, and experimental error.^22^ Calibration considered stroke length, direction, and speed of the movements that would be expected in a mastoidectomy.

## Results

### MARS Analysis

*T*-tests between models showed no significant differences in drill stroke frequency (cadaveric = 1.36/s, printed = 1.50/s, *P* < .40) but demonstrated significantly shorter duration (cadaveric = 0.37 s, printed = 0.16 s, *P* < .01) and a higher percentage of curved strokes (cadaveric = 31, printed = 67, *P* < .01) used in printed dissection procedures (Table 1).

**Table 1.** Analysis of Stroke Frequency and Linearity. Stroke duration was significantly shorter for printed bone model compared to cadaveric (P < .01) with a significantly higher percentage of curved strokes (P < .01).

	3D printed model	Cadaveric bone	*P*-value
Stroke frequency (strokes/s)	1.50	1.36	<.40
Stroke duration (s)	0.37	0.16	<**.01**
% Curved strokes	67	31	<**.01**

*Note.* Significant differences were present in stroke duration, and linearity of strokes.

**Figure 3.**
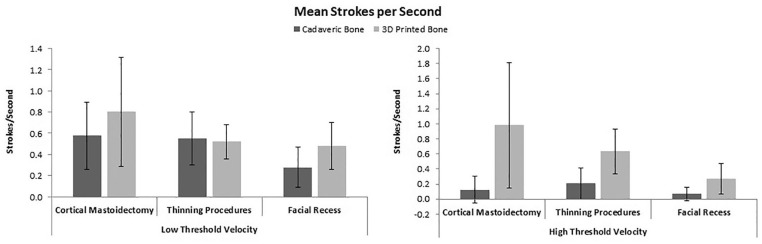
Comparison of mean strokes per second. Significant differences occur across high threshold velocity.

Strokes were determined both by (A) low threshold velocity and (B) high threshold velocity. Three (3) drilling segments observed were cortical mastoidectomy, thinning procedures, and facial recess. Comparison of low threshold stroke velocity between the printed and cadaveric conditions across cortical mastoidectomy, thinning procedures, and facial recess development found no difference, however there was significant differences in the high threshold velocity activities (*P* < .05) (Figure 3).

### K-Metric Analysis

**Figure 4.**
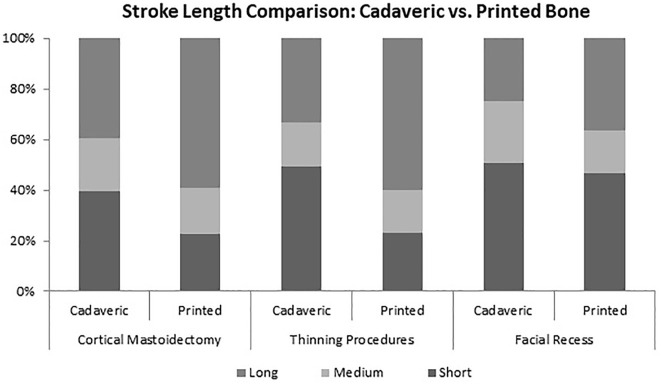
Stroke length comparison across conditions. Ratio of short (<5 mm) to medium (5-10 mm) to long strokes (>10 mm) for cadaveric compared to printed models for 3 drilling segments.

The k-cos software provided a greater ability to compare additional metrics such as ratio of straight strokes to curved strokes, individual stroke lengths, and time per stroke. A comparison of mean strokes per second was also completed with the k-cos analysis method. There were no significant differences between the cadaveric and printed model based on the sensor attached to the drill. There were significant differences found in the cortical mastoidectomy and thinning procedures with respect to short and long strokes (*P* < .05) (Figure 4).

**Figure 5.**
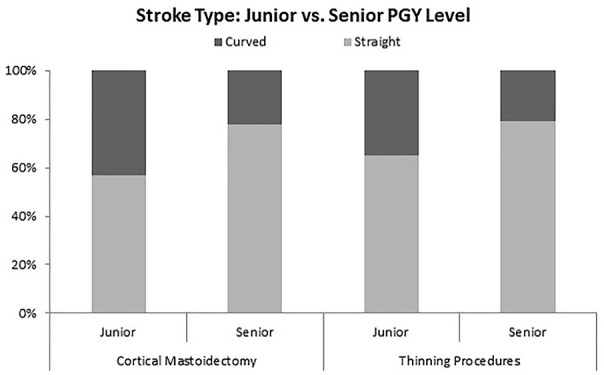
Comparison of junior and senior ratios of curved to straight strokes. Significance was noted with short strokes during thinning procedures.

**Figure 6.**
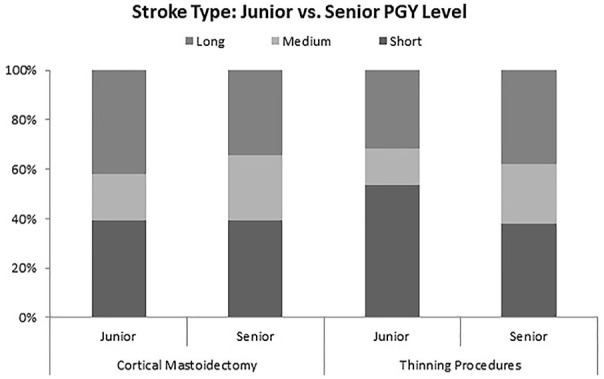
Ratio of short, medium, and long strokes made by junior compared to senior residents. All *t*-tests returned significant *P* values between junior and senior residents drilling technique.

In comparing junior with senior residents using this software, significant differences can be seen throughout the different metrics analyzed. Junior residents take a greater number of shorter strokes (Figure 5) during thinning procedures, as well as consistently using more curved strokes throughout the entire drilling session (Figure 6). Junior staff used a higher number of short strokes per second (junior = 0.54, senior = 0.38, *P* < .01) and higher percentage of curved strokes (junior = 35%, senior = 21%, *P* < .01).

## Discussion

The manner and environment in which residents are taught has a considerable impact on skill acquisition. Many articles espouse the potential benefits of all forms of simulated surgery. However, there is the possibility that these technologies may impair the development of surgical dexterous capacity. Augmenting the current paradigm with simulated models requires the diligence to assess the actual value in education.

Drilling technique can best be determined based on the drill tip performance, therefore analysis focused on this sensor. The sensor attached to the thumb was analyzed along that of the drill and consistently showed similar trends, though more muted. The reason behind this may be a result of the fact that farther from the drill tip there is less movement and thus becomes more difficult to distinguish a difference in the finer points of drilling technique.

An area of error is the thresholds set within the software. There is no way to eliminate noise without also removing finer intentioned movement. This may artificially elevate low threshold data. In this particular study, because the data is being compared within the same participant, it would be expected that if noise were present, it would be present throughout all samples, thus canceling out in the final analysis.

Further, the threshold limits for what constitutes a straight or a curved stroke is arbitrary. Had the limits been adjusted in either direction, the results would be demonstrably different. This is a very important consideration in the interpretation of this data.

### Limitations

This study was undertaken as a sample of convenience and lacked an expert control group for comparison. As such, a single center with a limited pool of participants may inhibit the generalizability of the findings.

### Comparison of Cadaveric Bone to 3D Printed Models

The analysis of the various metrics comparing cadaveric to printed modeling was challenging. Attempts to assess drill length, linearity, and frequency with employ of disparate sensor locations as well as both velocity and acceleration proved complicated.

Stroke duration was consistently shorter with the printed simulation. Hardness, elasticity, density, and other properties are important in how the drill burr removes material.^7^ As the printed models are strictly a representation of bone, the additional hindrances of soft tissue are not present. The role this assumes in enabling longer, faster strokes may be important. Moisture may also contribute to the difference in techniques, as irrigation was used in the cadaveric condition while no liquid was used during dissection of the printed.^23,24^

While significant differences in dissection of the 2 conditions are statistically present, an analysis of speed, safety, and the completeness of dissection was not undertaken. Both a formative and summative assessment of the trainee drill performance would have assisted in determining any relationship between drill technique and injurious activity. However, there is the strong possibility that trainees held a greater regard for the cadaveric model and acted accordingly with provision of greater focus and time.

Interestingly the printed model produced not only longer strokes but more rapid strokes as well. This may indicate that residents are less cautious when drilling the printed models or they are more able to appreciate anatomic areas of interest with the distinct coloration intrinsic to the model used. However, it is possible that these differences may be attributed to the mechanical properties of the materials.

### Comparison of 3D Printed Models to Haptic Models

The study completed by Ioannou et al^13^ found a number of differences between cadaveric bone and haptic models. Significant results were noted in strokes per second, mean stroke duration, mean stroke distance, and percentages for straight and curved strokes.^13^ Using the results from this k-cos analysis, the printed models showed differences in many of the same areas, though often of the opposite effect. More straight strokes were used with the haptic simulation, while more curved strokes were used with the printed model, when compared to cadaveric drilling. The same effect was also seen with strokes per second and stroke distance.

### Comparison of Junior to Senior Residents

Junior and senior residents showed significant differences when compared across domains. This indicates that there is indeed a learning process as drilling technique is developed as resident graduate to more senior levels.

### Summative Experience

Ultimately, we find differences in drill technique, yet are not positioned to determine the significance as the results are to a great extent dependent on software thresholding values. At this time it may be most productive to conceptualize both virtual and printed simulations as antecedent to cadaveric surgical rehearsal. The tools used to teach junior residents need to help initially with anatomic understanding, advancing to surgical capacity and then resolving difficult anatomy and pathology.

## Conclusions

Defining differences across training platforms is important in order to prevent maladaptive skills, particularly in junior residents who may not have yet developed a consistent technique. We found an increase in speed, length, and a reduction in linearity of trainee drill technique when using a printed model. Printed temporal bone models should be employed in the collective of disparate simulations to maximize access and ensure no singular modality unduly produces maladaptive technique.

Secondary findings showed significant differences between the drilling methods of junior residents compared to their senior counterpart, demonstrating evolution in technique during training.
